# The Effects of a Macromolecular Charring Agent with Gas Phase and Condense Phase Synergistic Flame Retardant Capability on the Properties of PP/IFR Composites

**DOI:** 10.3390/ma11010111

**Published:** 2018-01-11

**Authors:** Hongda Chen, Jihui Wang, Aiqing Ni, Anxin Ding, Xia Han, Ziheng Sun

**Affiliations:** 1 School of Materials Science and Engineering, Wuhan University of Technology, 122 Luoshi Road, Wuhan 430070, Hubei, China; Hongdachen@whut.edu.cn (H.C.); jhwang@whut.edu.cn (J.W.); hanxia@whut.edu.cn (X.H.); Sunziheng@whut.edu.cn (Z.S.); 2 State Key Laboratory of Advanced Technology for Materials Synthesis and Processing, Wuhan University of Technology, 122 Luoshi Road, Wuhan 430070, Hubei, China; 3 Lehrstuhl füCarbon Composites, Technische Universitaet Muenchen (TUM), 80333 Munich, Germany; axding@whut.edu.cn

**Keywords:** intumescent flame retardant, polypropylene, mechanical properties, thermal properties, flame retardancy mechanism

## Abstract

In order to improve the efficiency of intumescent flame retardants (IFRs), a novel macromolecular charring agent named poly(ethanediamine-1,3,5-triazine-p-4-amino-2,2,6,6-tetramethylpiperidine) (PETAT) with gas phase and condense phase synergistic flame-retardant capability was synthesized and subsequently dispersed into polypropylene (PP) in combination with ammonium polyphosphate (APP) via a melt blending method. The chemical structure of PETAT was investigated by Fourier transform infrared spectroscopy (FTIR), and ^1^H nuclear magnetic resonance (NMR) spectroscopy. Thermal properties of the PETAT and IFR systems were tested by thermogravimetric-derivative thermogravimetric analysis (TGA-DTG) and thermogravimetry–Fourier transform infrared spectroscopy (TG-FTIR). The mechanical properties, thermal stability, flame-retardant properties, water resistance, and structures of char residue in flame-retardant composites were characterized using tensile and flexural strength property tests, TGA, limiting oxygen index (LOI) values before and after soaking, underwritten laboratory-94 (UL-94) vertical burning test, cone calorimetric test (CCT), scanning electron microscopy with energy dispersive X-ray spectrometry (SEM-EDXS), and FTIR. The results indicated that PETAT was successfully synthesized, and when the ratio of APP to PETAT was 2:1 with 25 wt % loading, the novel IFR system could reduce the deterioration of tensile strength and enhance the flexural strength of composites. Meanwhile, the flame-retardant composite was able to pass the UL-94 V-0 rating with an LOI value of 30.3%, and the peak of heat release rate (PHRR), total heat release (THR), and material fire hazard values were considerably decreased compared with others. In addition, composites also exhibited excellent water resistance properties compared with traditional IFR composites. SEM-EDXS and FTIR analyses of the char residues, as well as TG-FTIR analyses of IFR were used to investigate the flame-retardant mechanism of the APP/PETAT IFR system. The results indicated that the efficient flame retardancy of PP/IFR composites could be attributed to the synergism of the free radical-quenching and char layer-protecting mechanisms in the gas phase and condense phase, respectively.

## 1. Introduction

Polypropylene (PP) has been widely used in various fields such as the automobile industry, appliances, and in electric shell and packaging materials, etc. [1,2,3]. This is largely due to its excellent mechanical properties, as well as its easy processing, low cost, and good chemical resistance. However, the neat PP resin cannot meet all requirements because of its low flame retardancy and poor thermal resistance properties. Introducing flame retardant into the PP matrix has been considered a cost-effective route for enhancing the flame retardancy and thermal stability of PP [4,5,6].

Halogenated flame retardants are not able to be applied to materials in many fields, even though they have highly efficient flame retardancy with a gas phase flame-retardant mechanism during polymer combustion [7,8,9]. This is because researchers have certified that halogens and their derivatives are toxic and carcinogenic to humans. Among all flame retardants, intumescent flame retardants (IFRs) have attracted considerable attention from researchers due to their low smoke, halogen-free, anti-dropping, environmental-friendly properties. An IFR system is usually composed of three components: an acid source, a charring source, and a gas source [10,11,12]. During the combustion of the PP/IFR composites, IFR can involve PP in a series of chemical reactions to form a compact and continuous intumescent carbonaceous layer which acts as a barrier to protect the inside material from combustion with a condense phase flame-retardant mechanism [13,14].

Ammonium polyphosphate/melamine/pentaerythritol (APP/MEL/PER) is one of the most used traditional IFR systems but this system has the disadvantages of poor flame-retardant efficiency, low thermal stability, and weak water resistance due to the low molecular weight of PER [15]. Synthesizing macromolecular charring agents to replace the small ones have been considered as an efficient way to conquer the problems listed above. The previous studies showed that macromolecular triazine-derived charring agents combined with APP could remarkably enhance flame-retardant properties as well as thermal stability and water resistance [13,16,17,18].

Considering the good quenching ability of free radicals, studies have focused on N-alkoxy hindered amines (NORs) with a synergistic effect in combination with IFRs to improve the flame-retardant properties of PP/IFR composites [19,20]. However, most of the relevant works introduced the NOR agents into the PP matrix through a mechanical blend method, which could cause the low molecular weight NORs to become unstable due to the high processing temperature [21]. Introducing the hindered amine group into macromolecular charring agent by covalent bonds is a good way to overcome these shortcomings [22].

In this paper, a triazine-derived macromolecular charring agent named poly(ethanediamine-1,3,5-triazine-p-4-amino-2,2,6,6-tetramethylpiperidine) (PETAT) with free radical-quenching capability was synthesized and subsequently dispersed into PP in combination with APP via a melt blending method. The chemical structure of PETAT was investigated by Fourier transform infrared spectroscopy (FTIR), and ^1^H nuclear magnetic resonance spectroscopy (^1^H NMR). The thermal properties of PETAT and IFR systems were tested by thermogravimetric-derivative thermogravimetric analysis (TGA-DTG) and thermogravimetry–Fourier transform infrared spectroscopy (TG-FTIR). The mechanical properties, thermal stability, flame-retardant properties, water resistance, and structures of char residue in flame-retardant composites were characterized by tensile and flexural strength property tests, TGA, limiting oxygen index (LOI) values before and after soaking, underwritten laboratory-94 (UL-94) vertical burning test, cone calorimetric test (CCT)), scanning electron microscopy with energy dispersive X-ray spectrometry (SEM-EDXS), and Fourier transform infrared spectroscopy (FTIR).

## 2. Experiments

### 2.1. Materials

PP (KP503), a granulated product with a melt flow index of 60.0 g/10 min (230 °C, 2.16 kg), was supplied by China Petroleum and Chemical Corporation, Beijing, China. NaOH, APP (polymerization degree >1500), PER, and ethylenediamine were purchased from Sinopharm Chemical Reagent Co. Ltd., Shanghai, China. Cyanuric chloride (CNC) was obtained from Shanghai Macklin Biochemical Co., Ltd., Shanghai, China. Triacetonediamine was obtained from Shanghai Aladdin Bio-Chem Technology Co., Ltd., Shanghai, China. All materials were used directly without further purification.

### 2.2. Synthesis of PETAT

Firstly, 27.66 g of CNC (0.15 mol) and 200 mL of 1,4-dioxane were added to a 500-mL round-bottom flask and stirred at 0–5 °C until a transparent solution was formed. Next, 23.44 g (0.15 mol) of triacetonediamine was dissolved into another 50 mL of 1,4-dioxane. Then, the mixture and NaOH aqueous solution were added dropwise into the CNC solution within 2 h. The pH value of the solution was kept at 5–6. The reaction lasted for 4 h at 0–5 °C.

After that, the temperature was raised to 40–50 °C; 5 mL (0.075 mol) of ethylenediamine and 3 g (0.075 mol) of NaOH mixture water aqueous solution were dropped into the flask within 2 h. The mixture was stirred for another 4 h with a pH value of 7–8. The solution was filtered and washed several times in deionized water, and then the intermediate 2,4-dichloro-1,3,5-triazine-p-4-amino-2,2,6,6-tetramethylpiperidine (DTAT) was obtained (yield: 91%).

**Figure 1:**
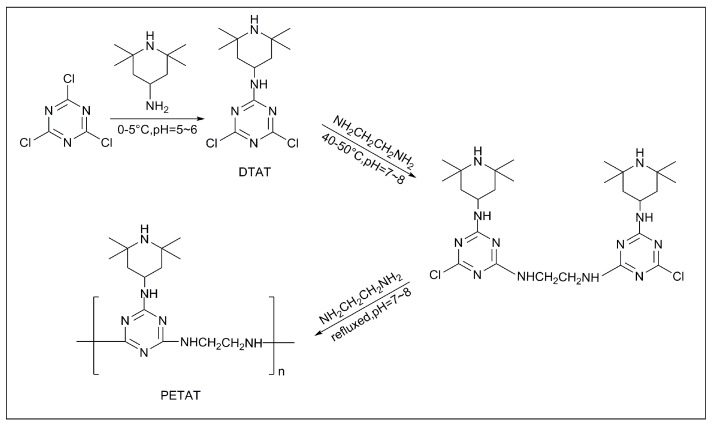
The synthesis route of TAT and PETAT (poly(ethanediamine-1,3,5-triazine-p-4-amino-2,2,6,6-tetramethylpiperidine)).

Thereafter, 43.65 g (0.15 mol) of DTAT and 200 mL of 1,4-dioxane were added to a 500-mL round-bottom flask and stirred until a transparent solution was formed. The mixture was heated to 100 °C with refluxing. Then, 5 mL (0.075 mol) of ethylenediamine and 3 g (0.075 mol) of NaOH mixture water aqueous solution were dropped into the flask within 2 h. The reaction lasted for 6 h with a pH value of 7–8. Then, the solution was filtrated and washed several times using deionized water. The products were dried under a vacuum at 80 °C for 12 h and the novel charring agent PETAT was obtained (yield: 89.6 %). The synthesis route is shown in Figure 1.

### 2.3. Preparation of PP Composites

The PP, APP, PETAT, and PER were dried in a vacuum oven at 80 °C for 12 h before use. All the composites were prepared on a two-roll mixing mill (Changzhou Suyan science and Technology Co., Ltd., Changzhou, China) at 180 °C for 10 min. The prepared mixtures were compressed and molded into standard samples for tests. The formulations of the flame-retardant PP composites are listed in Table 1.

### 2.4. Characterization

FTIR was performed using a Nexus infrared spectrometer (Thermo Nicolet 6700, Waltham, MA, USA) with thin films of KBr at room temperature. The measurement was carried out in the optical range of 4000–500 cm^−1^.

^1^H NMR spectrometry samples were recorded on a Fourier transform superconducting magnetic resonance spectrometer (Avance III HD 500 MHz, Bruker Inc., Bremen, Germany) using D_2_O as a solvent.

The tensile strength and flexural strength of PP and flame-retardant PP composites were measured at ambient temperature using an Instron universal testing machine (4302, Instron Corporation, UK), according to American Society for Testing and Materials D638 (ASTM D638, crosshead speed 10 mm/min) and ASTM D790 (in a three-point loading mode), respectively.

The TG-FTIR instrument consists of a thermogravimeter (STA449F3, Netzsch Instruments Co., Selb, Germany), and a Fourier transform infrared spectrometer (Thermo Nicolet 6700, USA). The investigation was carried out from 30 °C to 900 °C at a linear heating rate of 20 °C/min under a nitrogen flow of 30 mL/min.

The TGA-DTG test was carried out using thermogravimeter (STA449F3, Netzsch Instruments Co., Selb, Germany) to analyze the thermal characteristics of the samples. The samples were heated and scanned over a temperature range from room temperature to 700 °C at a heating rate of 20 °C/min under a nitrogen atmosphere. The initial decomposition temperatures and the thermal degradation weight losses (formation of char) of the samples were recorded and analyzed.

LOI values were determined using an oxygen index instrument (COI, Motis Fire Technology Co., Ltd., Suzhou, China) on 130 mm × 6.5 mm × 3.0 mm bars, according to the International Organization for Standardization 4589-1984 (ISO 4589-1984) standard. The water resistance test was performed using the LOI test with samples immersed in distilled water at 70 °C and kept at this temperature for 168 h. The treated samples were subsequently taken out and dried in a vacuum oven at 70 °C to a constant weight.

The UL 94 vertical burning test was carried out using an instrument (PX-03-001, Phinix Analysis Instrument Co., Ltd., Suzhou, China) on 125 mm × 12.5 mm × 3.2 mm bars, according to ANSL/UL-94-2009.

The CCT was carried out by using a cone calorimeter (Fire Testing Technology Co., East Grinstead, UK) according to ISO 5660. Each specimen, measuring 100 mm × 100 mm × 3.0 mm, was wrapped in aluminum foil and exposed horizontally to an external heat flux of 35 kW/m^2^. The residues of the samples after the test were photographed using a digital camera (DSC-RX10 II, SONY Inc., Tokyo, Japan).

The morphology of the residue char was observed by SEM (JSM-IT300, JEOL Ltd., Tokyo, Japan). The surface of the residue char was sputter-coated with a conductive gold layer before observation. EDXS results of the upper surfaces of residue char for the flame-retardant PP were measured by EDXS.

## 3. Results and Discussion

### 3.1. Characterization of DTAT and PETAT

**Figure 2:**
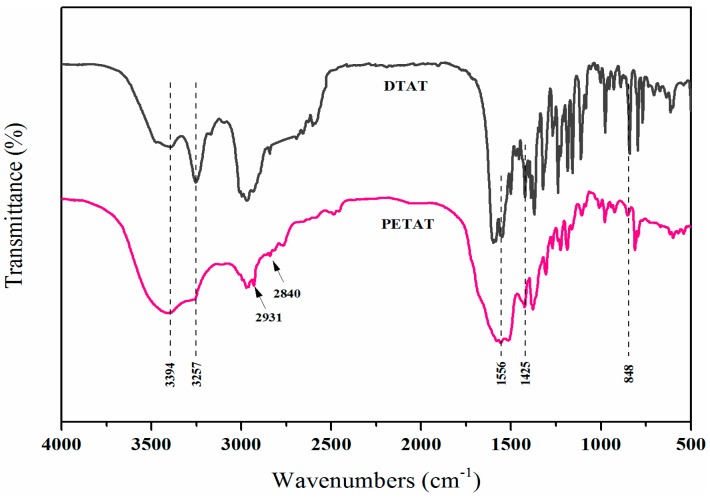
FTIR spectra of TAT and PETAT.

The FTIR spectra of DTAT and PETAT are presented in Figure 2. As for the DTAT and PETAT, N–H bands at 3394 cm^−1^ and 3257 cm^−1^ and C=N of triazine ring bands at 1425 cm^−1^ and 1556 cm^−1^ could be observed, which originated from tetramethylpiperidine and CNC, respectively. Compared with the spectrum of DTAT, two new peaks located at 2931 cm^−1^ and 2840 cm^−1^ (C–H of –CH_2_–) appeared and one peak at 848 cm^−1^ (C–Cl) disappeared in the PETAT spectrum, showing that a reaction had occurred between DTAT and ehanediamine and the Cl atoms had been replaced. The above results indicate that the PETAT was synthesized successfully.

**Figure 3:**
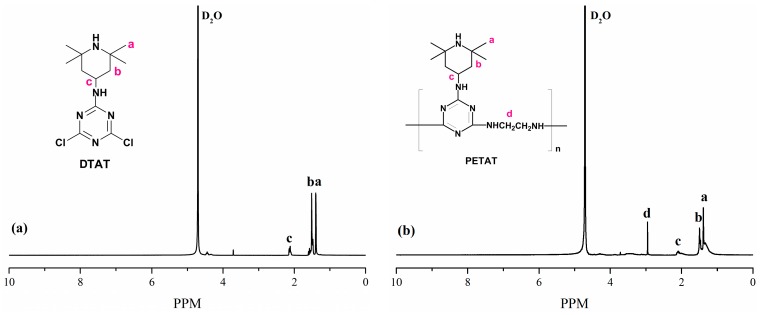
^1^H NMR spectra of (**a**) DTAT (2,4-dichloro-1,3,5-triazine-p-4-amino-2,2,6,6-tetramethylpiperidine) and (**b**) PETAT.

In order to further investigate the chemical structures of DTAT and PETAT, ^1^H NMR was used to analyze the varieties and states of H atoms in different groups. The spectra of DTAT and PETAT are shown in Figure 3. The signal at the chemical shift of 1.35–1.43 ppm was assigned to the H atoms of methyl group in the piperidine ring. The signals located at 1.44–1.58 ppm and 2.07–2.16 ppm were assigned to the H atoms of the methylene and methine groups, respectively [22]. The spectrum of PETAT contained all the characteristic signals of DTAT. Besides, a new signal located at 2.95 ppm appeared, which corresponded to the H atoms of the –CH_2_–CH_2_– group. All the information above indicates that the PETAT is successfully synthesized.

### 3.2. Mechanical Properties of Flame-Retardant PP Composites

**Figure 4:**
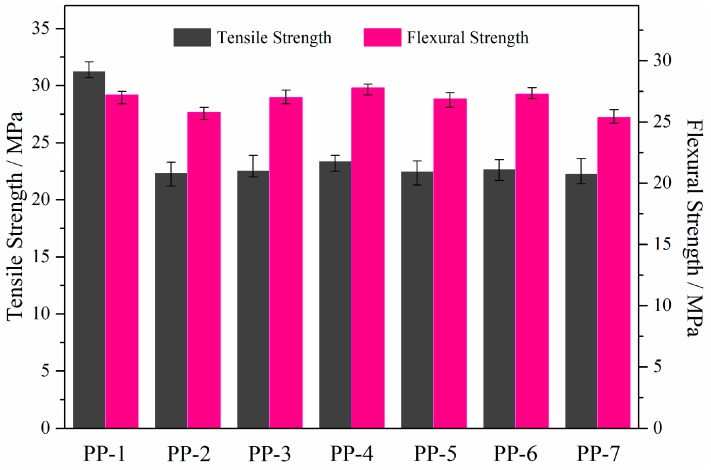
Tensile and flexural strength of PP and its composites.

Mechanical features such as the tensile strength and flexural strength properties of PP and its flame-retardant composites are shown in Figure 4. It was found that the tensile and flexural strength values of neat PP (PP1) were 31.32 ± 0.93 and 27.26 ± 1.26 MPa, respectively. Considering the typical error range of the tests, all formulations showed similar reductions in tensile strength, indicating that the incorporation of PETAT into PP matrix has a negative effect on the tensile property of composites. The results showed that PP/APP/PER composite (PP7) had lower mechanical properties than those of neat PP. The main reason is the low molecular weight of PER and the poor compatibility between the APP, PER, and PP matrix [16]. It was found that introducing the macromolecule charring agent PETAT into the PP matrix could improve the flexural strength to various degrees compared to samples without PETAT. When the IFR loading was 25 wt % and the ratio of w_APP_ to w_PETAT_ was 2:1, the PP/APP/PETAT composite (PP4) showed higher flexural strength properties compared to the others. This could be attributed to the lower polarity of PETAT, which acted as a coupling agent to improve the compatibility between the APP and PP macromolecules [16].

### 3.3. Thermal Properties and TG-FTIR Analysis

**Figure 5:**
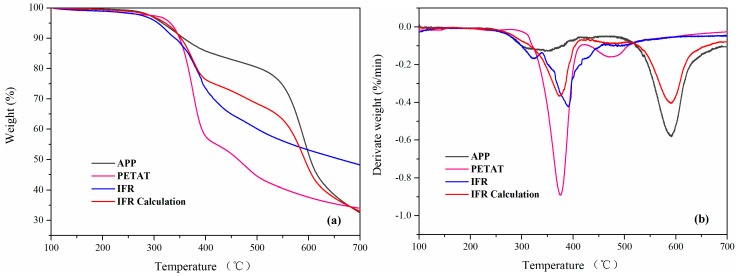
(**a**) TGA and (**b**) DTG (thermogravimetric-derivative thermogravimetric) curves of APP (Ammonium polyphosphate), PETAT, IFR (intumescent flame retardants), and IFR calculation under N_2_.

Figure 5 shows the TGA and DTG curves of APP, PETAT, and IFR (APP/PETAT, weight ratio was 2:1) as well as the IFR calculations under nitrogen conditions. The corresponding data are listed in Table 2. In the case of PETAT, it exhibited excellent thermal stability and its initial decomposition temperature (Ti, defined as the temperature at which 5.0 wt % mass loss occurred) was 331.6 °C, which could well meet the processing temperature for polypropylene. Meanwhile, the char residue content of PETAT was 33.9% at 700 °C, indicating that PETAT possessed outstanding char-forming ability and could be used as an efficient charring agent for the IFR system. The IFR curve calculations were performed using the experimental results and percentages of APP and PETAT according to Formula (1) [1,17]. Compared with the IFR calculation curve, the Ti of the IFR experimental curve was lower owing to phosphorylation, dehydration, and carbonization between APP and PETAT at a lower temperature [23]. With the increase of temperature, the surface char layer could protect the inner materials from further decomposition. Consequently, the T_50%_ (50 wt % mass loss) of the IFR experimental curve was greater than that of the calculated curve, and the char residue content in the IFR experimental curve at 700 °C was far greater than expected. Besides, the DTG curve of IFR calculation was classified into two steps while the IFR experimental curve showed only one step, which verified the synergistic effect between APP and PETAT.

(1) $$w_{\mathrm{calculation}}=w_{\mathrm{APP}}\times 66.7\%+w_{\mathrm{PETAT}}\times 33.3\%$$

**Figure 6:**
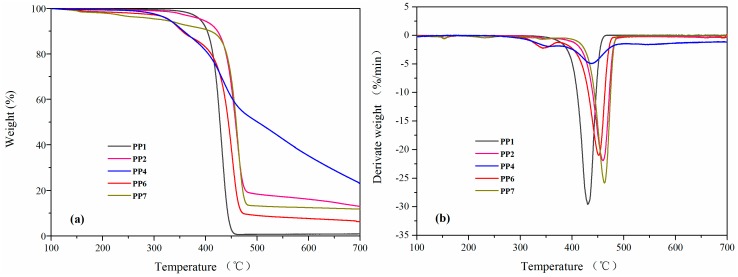
TGA (**a**) and DTG (**b**) curves of PP and PP composites under N_2._

Figure 6 shows the TGA and DTG curves of PP and flame-retardant PP under nitrogen conditions, and the detailed data are listed in Table 3. It can be seen that the neat PP (PP1) decomposed rapidly in the temperature range of 354.4 to 459.7 °C, with almost no residue left at 700 °C. Compared with neat PP and flame-retardant PP composites, the PP/APP/PER composite (PP7) showed a low T_i_ due to the low thermal stability of PER. When the APP/PETAT IFR system was introduced into the PP matrix, composites showed a lower T_i_ due to the decomposition and crosslinking reactions of IFR that occurred at a low temperature (307.2 °C) [24]. However, the T_50%_, T_max_ (maximum mass loss rate temperature), and char residue values of composites increased from 427.6 °C, 430 °C, and 0.9% for neat PP to 501.1 °C, 437.3 °C, and 23.0 % for the PP/APP/PETAT composites. Meanwhile, the maximum degradation rate (R_max_) of the PP/APP/PETAT (PP4) composite was significantly lower than for the others. The explanation could be that the synergistic effect between APP and PETAT promoted an intumescence char layer that formed before the PP matrix decomposed, which could act as thermal barrier to inhibit the transition of heat and flammable gas. All results indicated that the APP/PETAT IFR system could decrease the decomposition of the inside matrix and improve the thermal stability of PP [13,25].

**Figure 7:**
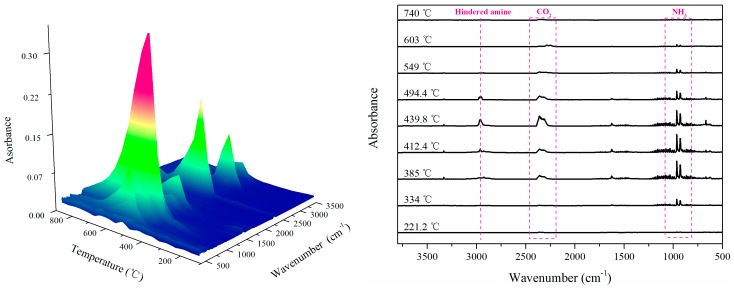
3D TG-FTIR spectra pyrolysis products for PETAT/APP (weight ratio is 2:1) and corresponding FTIR spectra at different temperature.

To further explore the synergistic mechanism of APP/PETAT IFR system, the gaseous pyrolysis products of IFR were analyzed using the TG-FTIR test. The 3D TG-FTIR spectra and the characteristic spectra obtained at different temperatures for APP/PETAT IFR (the mass ratio of APP to PETAT was 2:1) are presented in Figure 7. It can be seen that there was no obvious infrared absorption signal below 220 °C, revealing that no decomposition of IFR occurred, which could meet the need of melt blending for PP. At 334 °C, new peaks appearing at 927 cm^−1^ and 965 cm^−1^ are attributed to NH_3_ absorptions, illustrating the decomposition of APP. By heating to 385 °C, the absorptions of CO_2_ at 2277 cm^−1^–2395 cm^−1^ indicated the further decomposition of APP and PETAT [14]. Furthermore, the peak of nitroxyl radicals (2962 cm^−1^) generated from the thermal decomposition of PETAT was found at 412.4 °C. When the temperature increased to 439.8 °C, the maximum signal intensity was observed, which indicated that complicated reactions had occurred. The results indicated that the gaseous pyrolysis product-generated process closely matched the degradation of IFR. The absorption peaks of CO_2_ could be observed between 385 °C and 495 °C, which meant that the molten char layer formed at this temperature range [22]. Meanwhile, the maximum release rate of NH_3_ appeared at 412.4 °C, which could well match with the char-forming process, and formed an intumescent char layer; In addition, the hindered amine pyrolysis product with active free radical-quenching capability (nitroxyl radicals) could be detected between 412.4 °C and 494.4 °C, demonstrating that free radicals like H^•^ and OH^•^ could be quenched and the flame could be suppressed simultaneously [26].

### 3.4. Flame Retardancy and Water Resistance of Flame-Retardant PP Composites

**Figure 8:**
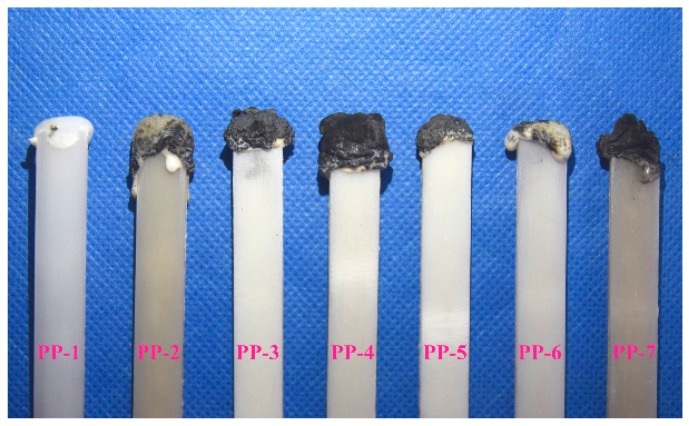
Photos of char residues after LOI test for PP and PP composites.

The LOI values before and after soaking, the UL-94 rating, and the dropping behavior are tabulated in Table 4. It was found that neat PP presented a low LOI value of 16.4 and no rating in the UL-94 test. PP-4 exhibited good flame-retardant properties, which were able to achieve a UL-94 V-0 rating, and its LOI value was 30.3 with the addition of a 25 wt % novel IFR system when the weight ratio of APP and PETAT was 2:1. Compared with the traditional IFR system APP/PER-modified PP (PP7), PP4 showed better flame-retardant properties, both before and after water soaking. During the combustion of PP4, the flame clearly flickered with a squeaking sound; this could be attributed to the nitroxyl radical decomposition by PETAT which could catch the free radicals and reduce the degree of combustion [22]. It can be concluded that PETAT obtains an excellent synergistic effect with APP, and the novel IFR system can promote the formation of a protective char layer (Figure 8) to improve the flame-retardant properties of PP composites, like other methods used in research [5,6].

**Figure 9:**
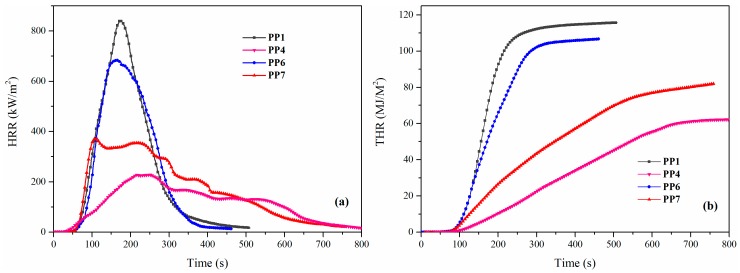
Heat release rate (**a**) and total heat release (**b**) curves of PP and PP composites.

The CCT was used to evaluate the flammability properties of PP composites and further explain the flame retardancy mechanism of the IFR system. Figure 9 shows the heat release rate (HRR) curves and total heat release (THR) curves of neat PP and its flame-retardant composites. The characteristic parameters of time to ignition (TTI), time to peak of heat release rate (TPHRR), peak of heat release rate (PHRR), THR, fire performance index (FPI = TTI/PHRR), and residual weight are listed in Table 5. The results were obtained at a heat flux of 35 kW/m^2^.

The TTIs of PP4 (28 s), PP6 (30 s), and PP7 (32 s) were shorter than those of the neat PP (41 s), due to the lower decomposition temperature of IFR systems compared with the PP matrix. Compared with PETAT and APP/PER IFR system, APP/PETAT IFR exhibited a higher efficiency with respect to the flame retardancy properties in PP composites. With the addition of the APP/PETAT IFR system into the PP matrix, the PHRR value decreased from 840.3 kW/m^2^ to 227.9 kW/m^2^ for neat PP, while the PP/APP/PER composite (PP7) exhibited a higher value of 354.7 kW/m^2^. This was due to the char layer being formed, which could prevent the PP matrix from decomposing. Furthermore, the HRR curve (Figure 9a) of the PP/APP/PETAT composite (PP4) showed several peaks in the rate of heat release, which is in accordance with the typical behavior of highly efficient IFR systems [17,27].

The THR curves of the PP and flame-retardant PP composites are shown in Figure 9b. The slope of the THR curve was considered to be representative of the trend of fire spread [28,29]. It was found that neat PP (PP1) released a total heat of 115.7 MJ/m^2^ and the PP/APP/PER composite (PP7) released 82.0 MJ/m^2^, whereas only 62 MJ/m^2^ was released by the PP/APP/PETAT composite (PP4). This indicated that the char layer of the PP/APP/PER composite (PP4) acted as insulating barrier to protect the substrate resin from heat and flame [30]. FPI was defined by the ratio of the TTI value to the PHRR value, which was chosen to further evaluate the fire hazard of the samples, and greater FPI values signified a lower fire hazard of the materials [31]. The PP/APP/PETAT composite (PP4) possessed the highest value for the FPI, which indicated it was the composite with the lowest fire hazard. The char residue values revealed that a more intumescent char layer was formed when the APP/PETAT IFR system was added. All results indicate that the APP/PETAT IFR system has excellent flame retardancy properties for the PP composite.

### 3.5. Morphological and Chemical Analysis of Residues

**Figure 10:**
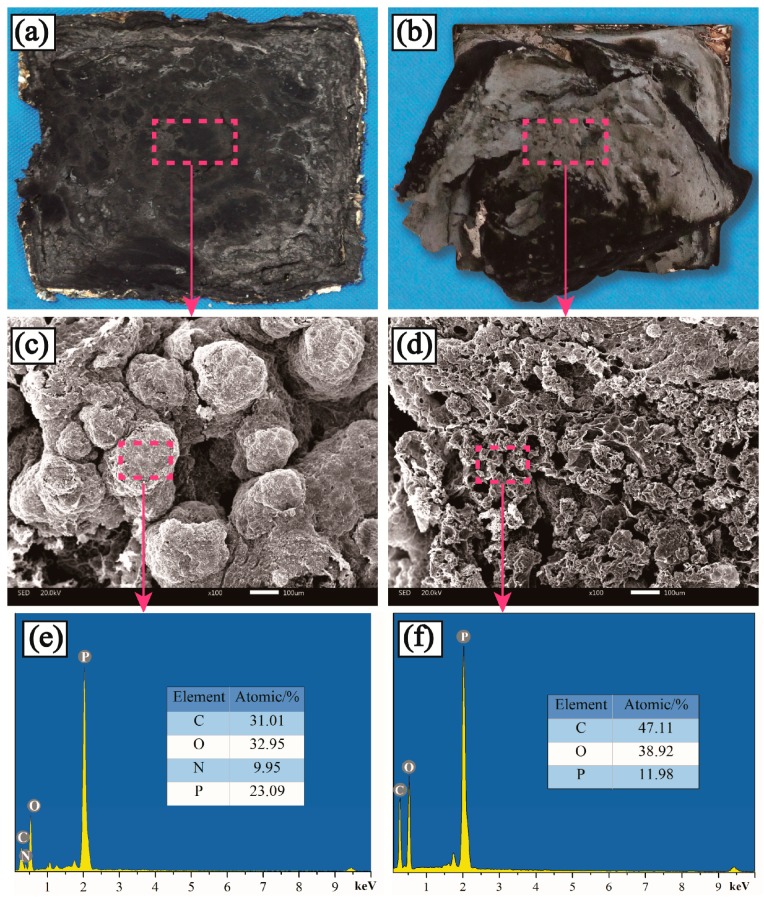
Photos, SEM (scanning electron microscopy) images and chemical compositions of residue char for PP4 and PP7.

The macrographs and the SEM-EDXS test for the residues of PP4 and PP7 after CCT are presented in Figure 10a–f, respectively. In the case of PP7, there were some microholes on the surface of the char layer which was loose and easy to break (Figure 10b), while the char layer of PP4 was continuous and compact with no visible holes on the surface (Figure 10b). This efficiently hindered the heat transmission and gas diffusion. The relationship between the microstructure of intumescent char layer and the flame-retardant properties of composites was further explained by the results of SEM (Figure 10c,d). It could be found that the char layer surface for the PP7 was full of holes which could hardly prevent heat and combustible gases from transferring. On the contrary, the surface of char layer for PP4 was almost completely covered with a swollen and compact char layer which could act as a barrier shield to protect the substrate from combustion [32]. The chemical composition of the char layer was analyzed by EDXS (Figure 10e,f). It was obvious that PP4 possessed higher P and N contents than PP7, indicating that the triazine-ring of PETAT could be involved in the crosslink action with P–O–C and P–O–N groups to form a more compact and thicker char layer [33].

**Figure 11:**
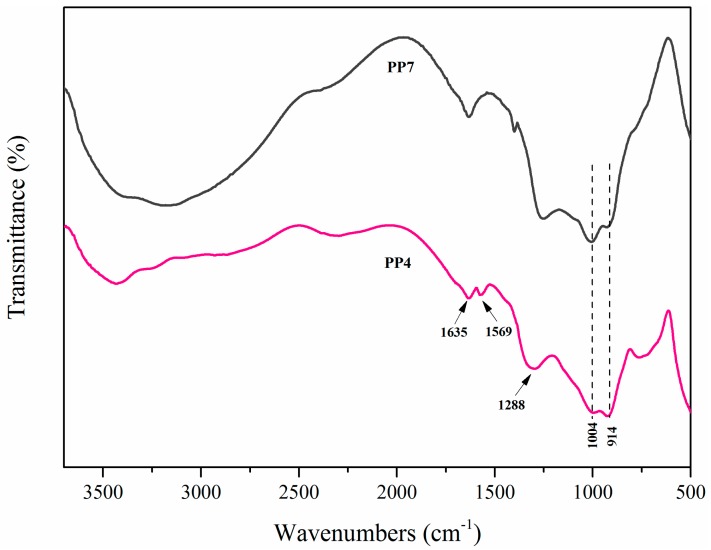
FTIR spectra of PP4 and PP7 residue char.

FTIR was employed to further examine the chemical compositions of char residuals of PP4 and PP7 after CCT, and the spectra are presented in Figure 11. As for PP4, bands around 914 cm^−1^, 1004 cm^−1^, 1569 cm^−1^, 1288 cm^−1^, and 1635 cm^−1^ were attributed to P–O–P, P–O–C, the triazine ring, and P=O and C=O structures, respectively [17,22]. Comparing the two spectrums, the main difference was the existence of a band at 1569 cm^−1^ in PP4, reflecting the stretching of the triazine ring. The test results indicate that the cross-linking reaction that occurred between APP and PETAT and the triazine ring can help to form an insulating barrier to prevent the heat transfer between the flame zone and the underlying substrate and protect the substrate from heat and fire.

### 3.6. Flame-Retardant Mechanism

**Figure 12:**
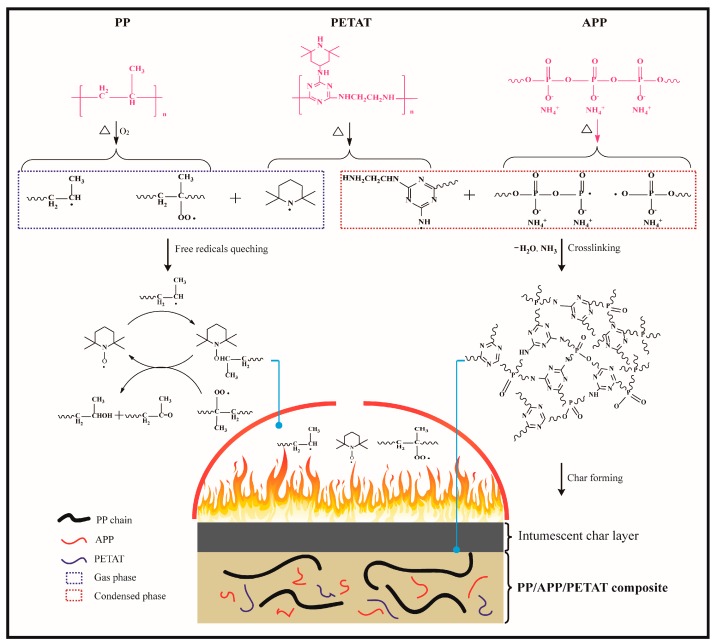
Flame retardant mechanism of PETAT/APP IFR system in PP matrix.

Based on the analysis of TGA, TG-FTIR, SEM-EDXS, and FTIR tests, the flame-retardant mechanism of the PETAT/APP IFR system in the PP matrix is illustrated in Figure 12. The charring agent PETAT could involve the IFR system in both a gas phase and a condensed phase flame-retardant mechanism. In the gas phase, free radicals such as those of the β-scission of PP chains could accelerate the degradation of the PP matrix [34]. The nitroxyl radicals produced by the thermal decomposition of PETAT could catch the free radicals produced by PP during the flame process and reduce the combustible gas by converting the free radicals into stable alcohols and ketones [19,35]. In the condensed phase, triazine oligomers radicals decomposed from PETAT, and phosphorus-containing compounds (phosphate ester radicals and pyrophosphate radicals) produced by APP could be involved in the free radical chemical reactions and form a crosslinking precursor char. Meanwhile, incombustible gases such as NH_3_ and H_2_O were released by APP and swelled the precursor char. As the temperature increases, a high-quality char layer could form and act as a barrier to prevent the PP matrix from further decomposing.

## 4. Conclusions

In order to address the issue of low efficiency of intumescent flame retardants (IFRs), a novel charring agent PETAT with gas phase and condense phase synergistic flame-retardant capability was synthesized, and flame-retardant PP composites were fabricated using a melt blending method. The 25 wt % novel IFR system loading (weight ratio of APP to PETAT of 2:1) could endow the PP composite with better properties than the traditional one. The macromolecule charring agent PETAT was able to act as a coupling agent which decreased the deterioration of tensile strength and enhanced the flexural strength of composites. The thermal stability of novel IFR-modified PP composites was improved to a certain extent. Compared with the traditional IFR system (APP/PER), the APP/PETAT IFR system exhibited greater flame retardancy and a more water-resistant effect on PP, shown through higher LOI values and UL-94 V-0 rating, lower PHRR and THR values, and reduced fire hazard and deterioration of LOI values after water soaking. TG-FTIR and FTIR indicated that the efficient flame retardancy of PP/APP/IFR composites could be attributed to the synergism of the protective char layer and free radical-quenching mechanism. The novel flame-retardant system in this study has potential applications as a highly efficient flame retardant in the PP matrix, respecting the environment and human health. Due to the good suitability of PETAT, which was used in the high melting index PP matrix (60.0 g/10 min), our approach can also find potential applications in flame retardancy of continuous fiber-reinforced PP composites, which are highly attractive for military and civil infrastructure applications.

## Figures and Tables

**Table 1:** Formulations for flame retardancy PP (Polypropylene) composites.

Samples	PP (wt %)	APP (wt %)	PETAT (wt %)	PER (wt %)
PP1	100	0	0	0
PP2	75	25	0	0
PP3	75	18.7	6.3	0
PP4	75	16.6	8.4	0
PP5	75	12.5	12.5	0
PP6	75	0	25	0
PP7	75	18.7	0	6.3

**Table 2:** TGA and DTG data of APP, PETAT, IFR, and IFR calculation under N_2_.

Samples	*^c^* T_i_ (°C)	*^c^* T_50%_ (°C)	*^d^* T_max_ (°C)	*^e^* Residue (%)
APP	316.3	603.5	591.6	32.5
PETAT	331.6	463.4	375.3	33.9
*^a^* IFR	307.2	662.7	389.8	48.2
IFR Calculation *^b^*	321.9	592.4	590.1	32.9

*^a^* IFR is composed by APP and PETAT, (APP/PETAT, weight ratio is 2:1). *^b^* $w_{\mathrm{calculation}}=w_{\mathrm{APP}}\times 66.7\%+w_{\mathrm{PETAT}}\times 33.3\%$. *^c^* T_i_ and T_50%_ are the temperature at 5 wt % and 50 wt % mass loss, respectively. *^d^* T_max_ is the maximum mass loss rate temperature. *^e^* Char residue at 700 °C.

**Table 3:** TGA and DTG data of PP and PP composites under N_2_.

Samples	*^a^* T_i_ (°C)	*^a^* T_50%_ (°C)	*^b^* T_max_ (°C)	*^c^* R_max_ (%/min)	*^d^* Residue (%)
PP1	390.0	427.6	430	29.5	0.9
PP2	394.2	457.9	459.9	21.9	13.0
PP4	328.8	501.1	437.3	4.9	23.0
PP6	329.8	444.0	451.2	21.0	6.3
PP7	317.6	459.1	462.8	25.8	11.8

*^a^* T_i_ and T_50%_ are the temperature at 5 wt % and 50 wt % mass loss, respectively. *^b^* T_max_ is the maximum mass loss rate temperature. *^c^* R_max_ is the maximum decomposition rate. *^d^* Char residue at 700 °C.

**Table 4:** Before and after soaking LOI (limiting oxygen index) and UL-94 vertical test of PP and pp composites.

Samples	LOI (%)	UL-94 Rating	Dropping	After Soaking LOI (%)
PP1	16.4	No rating	Yes	16.4
PP2	21.2	No rating	Yes	19.1
PP3	28.1	No rating	Yes	25.6
PP4	30.3	V-0	No	27.8
PP5	28.4	V-1	No	26.6
PP6	17.8	No rating	Yes	16.9
PP7	24.7	V-1	No	19.4

**Table 5:** Characteristic parameters of the CCT (cone calorimetric test) for PP and PP composites.

Samples	TTI (s)	TPHRR (s)	PHRR (kW/m^2^)	THR (MJ/m^2^)	FPI (s·m^2^/kW)	Residue (wt %)
PP1	41	174	840.3	115.7	0.0488	1.4
PP4	28	250	227.9	62.0	0.1229	43.5
PP6	30	164	684.0	106.7	0.0439	5.8
PP7	32	218	354.7	82.0	0.0902	29.8
